# Effect of the inoculation of plant growth-promoting rhizobacteria on the photosynthetic characteristics of *Sambucus williamsii* Hance container seedlings under drought stress

**DOI:** 10.1186/s13568-019-0899-x

**Published:** 2019-10-31

**Authors:** Fangchun Liu, Hailin Ma, Lin Peng, Zhenyu Du, Bingyao Ma, Xinghong Liu

**Affiliations:** 1 Institute of Resource and Environment, Shandong Academy of Forestry, 42 Wenhua East Road, Jinan, 250014 Shandong People’s Republic of China; 2 Shandong Engineering Research Center for Ecological Restoration of Forest Vegetation, Jinan, 250014 Shandong China

**Keywords:** Plant growth-promoting rhizobacteria, *Acinetobacter calcoaceticus*, Photosynthetic, Drought stress, *Sambucus williamsii* Hance

## Abstract

Plant growth-promoting rhizobacteria (PGPR) are beneficial bacteria that survive within the range of plant rhizosphere and can promote plant growth. The effects of PGPR in promoting plant growth, activating soil nutrients, reducing fertilizer application, and improving the resistance of plant inducible system have been widely investigated. However, few studies have investigated PGPR as elicitors of tolerance to abiotic stresses, especially drought stress. In this study, the effects of *Acinetobacter calcoaceticus* X128 on the photosynthetic rate (*P*_n_), stomatal conductance (*G*_s_), intracellular CO_2_ concentration (*C*_i_), and total chlorophyll content [Chl(a+b)] of *Sambucus williamsii* Hance seedling leaves under moderate drought stress and drought-rewatering conditions were determined. Compared with those of uninoculated seedlings, the average *P*_n_ values during the entire drought stress of inoculated seedlings increased by 12.99%. As the drought duration was lengthened, *C*_i_ of uninoculated leaves continued to increase after rapidly declining, whereas *G*_s_ continuously decreased. Furthermore, their photosynthetic properties were simultaneously restricted by stomatal and non-stomatal factors. After X128 inoculation, *C*_i_ and *G*_s_ of *S. williamsii* Hance leaves continued to decrease, and their photosynthetic properties were mainly restricted by stomatal factors. At the end of the drought stress, water stress reduced [Chl(a + b)] of *S. williamsii* Hance leaves by 13.49%. However, X128 inoculation decreased this deficit to only 7.39%. After water supply was recovered, *P*_n_, *G*_*s*_, and [Chl(a+b)] in uninoculated leaves were reduced by 14.23%, 12.02%, and 5.86%, respectively, relative to those under well-watered conditions. However, *C*_i_ increased by 6.48%. Compared with those of uninoculated seedlings, *P*_n_, *G*_*s*_, and [Chl(a+b)] in X128-inoculated seedlings were increased by 9.83%, 9.30%, and 6.85%, respectively. Therefore, the inoculation of X128 under arid environments can mitigate the reduction of chlorophyll, delay the restriction caused by non-stomatal factors to *P*_n_ in plant leaves under water stress, and can be more conducive to the recovery of photosynthetic functions of leaves after water supply is recovered.

## Introduction

Water stress is considered one important factor limiting worldwide agricultural productivity and efficiency (Vivien et al. 2017). Changes in global air temperature and climate are leading to longer drought periods and more extremely dry years (Cook et al. 2014; Miroslav et al. 2015). Climate change is predicted to decrease water availability and increase drought risk, which is one of the major agricultural problems reducing crop yield in an arid or semiarid area (Prudent et al. 2015; Ali et al. 2017). Under this context of climate change, plants will be more vulnerable to severe drought conditions (Kaushal and Wani 2016, Gaion et al. 2018). Because the leaf is the organ most responsive to environmental conditions (Nevo et al. 2000; Guerfel et al. 2009), its structure reflects the effects of water stress more clearly than the stem and roots. Drought triggers many leaf responses, especially stomatal closure that negatively impacts photosynthesis and crop yield under water-deficit conditions (Evans and Loreto 2000). The reduction of photosynthesis under water deficit can potentially be ascribed to a decrease in both stomatal and mesophyll conductance (Gaion et al. 2018). Furthermore, gas exchange in plants under drought stress is restricted by stomatal or non-stomatal factors with lowered photosynthetic rate, and plant growth and dry matter accumulation are severely impacted (Reed and Loik 2016). However, restricted by stomatal conductance under water stress, intracellular CO_2_ concentration cannot satisfy the demand for photosynthesis. This condition is called the stomatal restriction of photosynthesis. A non-stomatal restriction of photosynthesis occurs when chloroplast activity and ribulose-1,5-bisphosphate carboxylase activity are reduced and photosynthetic function is weakened (Rakocevic et al. 2018; Staniak et al. 2017). Under mild and moderate water stress conditions, stomatal restrictive factors can greatly affect photosynthesis. However, under severe water stress condition, the effect of non-stomatal restrictive factors on photosynthesis plays a dominant role (Bellasio et al. 2018).

Plant growth-promoting rhizobacteria (PGPR) are beneficial bacteria that survive within the range of plant rhizosphere (Rubin et al. 2017; Shi et al. 2019) and can promote plant growth (Arkhipova et al. 2005), activate soil nutrients (Liu et al. 2017; Freitas et al. 2019), reduce fertilizer application (Vessey 2003), and improve the resistance of plant inducible systems (Yang et al. 2009). PGPR play an important role in enhancing plant growth through various mechanisms. The action mode of PGPR that promotes plant growth includes (I) nutrient fixation for easy uptake by plant abiotic stress tolerance in plants; (II) plant growth regulators; (III) siderophore production; (IV) volatile organic compound production; and (V) the production of protection enzymes, such as chitinase, glucanase, and ACC-deaminase for the prevention of plant diseases (Choudhary et al. 2011; García-Fraile et al. 2015; Vejan et al. 2016). PGPR can produce phytohormones, especially cytokinin (CTK), which play an important role in PGPR-promoted plant growth (Ahmed and Hasnain 2010; Khan et al. 2014; Zhang et al. 2011). Liu et al. (2013) believed that PGPR can improve CTK synthesis, transportation, and redistribution to facilitate plant growth. CTK is also closely related to the stress resistance of plants (García de Salamone et al. 2001; Arkhipova et al. 2007). Wang and Huang (2004) found that synthesized 6-benzyl aminopurine can expedite the growth and physiological recovery of Kentucky bluegrass under arid environment. Research on grapes shows that zeatin and zeatin riboside are reduced by over 50% after drying treatment of their local root zones (Stoll et al. 2000). Following local drought stress treatment of tomato roots, zeatin riboside content in xylem is reduced (Kudoyarova et al. 2007). The concentrations of CTKs, such as zeatin and zeatin riboside, in binding form in the xylem juice of sunflower are reduced under drought stress (Hansen and Dörffling 2003). In addition, increasing CTK concentration can promote the opening of plant stomates (Vysotskaya et al. 2003). In view of relationships among PGPR, CTK, and plant drought resistance, the effect of CTK-producing PGPR on the photosynthetic properties of plants under drought stress must be explored.

*Sambucus williamsii* Hance is a deciduous tree or shrub that belongs to the *Caprifoliaceae* family. It is an important eco-functional plant integrating ornamental function and ecological restoration with extremely strong environmental adaptability, anti-drought and anti-barren properties, resistance against diseases and pests, stability, and high yield (Yang et al. 2016). Therefore, keeping in view the beneficial effects of PGPR, changes in the photosynthetic parameters of *S. williamsii* Hance leaves were determined under moderate drought stress to evaluate the effects of PGPR on plant photosynthetic characteristics in drought stress, with the aim to provide new insights into methods to improve the plant’s drought resistance ability.

## Materials and methods

### Preparation of X128 and its bacterial solution

The bacterial strain X128 used in the present investigation was isolated and screened from walnut rhizosphere in drought stress using the serial dilutions method and bioassay for cytokinin production method described by Hussain and Hasnain (2011). Characteristics of physiology and biochemistry of the bacterial isolate are shown in Table 1. On the basis of 16S rRNA sequencing data, the bacterial isolate showed 99.2% similarity with *Acinetobacter calcoaceticus* (AJ888983). X128 was identified as *A. calcoaceticus* and saved in the China General Microbiological Culture Collection Center as: CGMCC No.7071. The 16S RNA gene sequence was submitted to GenBank under Accession Number KC428748. *A. calcoaceticus* X128 was inoculated into LB culture medium and cultivated at 28 °C for 3 days with shaking at 180 r min^−1^. As determined by High Performance Liquid Chromatography, this bacterial strain can produce 368.73 ng mL^−1^ zeatin and 310.77 ng mL^−1^ kinetin (Table 1). The OD_600_ value of this bacterial suspension was determined via spectrophotometry. The bacterial suspension was centrifuged at 10,000 rpm for 10 min. The supernatant was obtained and added with isovolumetric Salkowski (50 mL of 35% HClO_4_ + 1 mL of 0.5 M FeCl_3_) color matching solution. The mixture was allowed to stand for 30 min in the dark and then its OD_530_ value was determined. The IAA content in the fermentation liquor within unit volume was determined under OD_600_ = 1. IAA content was calculated through the standard curve of IAA gradient dilution. X128 produced 26.71 μg (mL OD_600_)^−1^ of IAA, thus proving that this bacterial strain had a certain potential of promoting plant growth.

**Table 1:** Characteristics of physiology and biochemistry of *Acinetobacter calcoaceticus* X128

	IAA produced (μg mL^−1^)	Kinetin produced (ng mL^−1^)	Siderophore production	P solubility	N-fixation	Trans-zeatin produced (ng mL^−1^)	
Main ability	26.71 (3.71^a^)	310.77(32.67)	–	–	–	368.73 (31.26)	

	Glutamate transferase test	Citrate utilization test	Gas production of glucose	Gluten hydrolysis	Growth at 37 °C	Growth at 41 °C	Growth at 44 °C
Biochemical characteristics	+^b^	+	+	−	+	+	−

−: negative

^a^Values are standard deviations (*n* = 3)

^b^+: positive

### Plot culture experiment

The experiment was performed in the greenhouse of Shandong Academy of Forestry. Pot culture soil comprised moistened soil with 34.64% field moisture capacity, 28.36 mg kg^−1^ alkali-hydrolyzable nitrogen, 37.45 mg kg^−1^ available phosphorous, 65.44 mg kg^−1^ available potassium, and pH of 7.71. The pot was 25 cm in height and 30 cm in width. Each pot was filled with 12 kg of soil. A total of 108 pots were prepared, each of which was planted with one *S. williamsii* Hance container seedling with an average ground diameter of 3.82 ± 0.04 mm and average plant height of 23.55 ± 0.20 cm. After *S. williamsii* Hance container seedlings were planted, they were uniformly tended, and soil moisture content was maintained at 70–75% of field moisture capacity.

*A. calcoaceticus* X128 was inoculated into beef extract–peptone medium and shook below 37 °C at a rate of 180 r min^−1^ for 3 days. The culture was then transferred to fluid medium at 10% inoculum size and then cultivated below 30–35 °C for 12 h with shaking at 180 r min^−1^. After viable bacterial count was determined, the culture was diluted to 2 × 10^8^ cfu mL^−1^ using distilled water. On May 20, 2018, 72 pots were randomly selected for X128 inoculation. Approximately 25 mL of X128 bacterial solution was diluted to 250 mL, and then uniformly irrigated within the X128-treated *S. williamsii* Hance seedling rhizosphere. On June 24, 36 pots of X128-inoculated *S. williamsii* Hance seedlings were selected, and watering of these *S. williamsii* Hance seedlings together with 36 pots of non-X128-inoculated seedlings was stopped. On June 29 (40 days after X128 inoculation), the soil was naturally dried to 43.8% of field moisture capacity and then subjected to drought stress test. Soil moisture content in the well-watered treatment and that under drought stress was maintained at 70–75% and 40–45% of field moisture capacity, respectively. Soil moisture content was measured using a portable moisture meter (model HH2, Delta-t Devices LTD, Burwell, UK) with a wet sensor (Type Wet-2-K1, Delta-t Devices LTD, Burwell, UK) at 9:00 am each day. After soil moisture contents under drought stress treatment and X128 inoculation treatment reached the standard, water was occasionally supplemented, and soil moisture content was maintained relatively stable. On August 5, water supply was recovered for the two drought stress treatments until soil moisture content reached the control level.

### Photosynthetic determination

Photosynthetic parameters were determined for the first time on May 20, 2018 at 10:00–10:30. A LI-6400 portable photosynthesis apparatus (LI-COR, USA) was used to determine the photosynthetic rate, stomatal conductance, and intracellular CO_2_ concentration of leaves. Three seedlings were randomly selected, and three healthy functional leaves of each seedling were selected with the same leaf position. Photosynthetic determination of each leaf was conducted for three times. After June 29, 2018, related photosynthetic indicators were determined every 6 days until the drought stress test ended on August 4, 2018. Leaves under different treatments were obtained at 1, 40, 50, 60, and 76 days after inoculation to measure chlorophyll content [Chl(a+b)] for three times. [Chl(a+b)] in the leaves was determined using the 96% ethanol immersion method (Ying et al. 2015). A total of 0.1 g of leaves from six seedlings was cut into small pieces (0.2 cm filaments) and extracted with 8 mL of 95% (v/v) alcohol in the dark for 24 h at 25 °C until the leaves were blanched. The absorbance of the supernatant was measured at 645 and 663 nm with a Shimadzu UV-2550 spectrophotometer (Kyoto, Japan). Chlorophyll concentrations were calculated by the standard method of Arnon (1949) and expressed as mg g^−1^ fresh weight (FW). On August 11 (6 days after the recovery of water supply), the photosynthetic rate, stomatal conductance, intracellular CO_2_ concentration, and [Chl(a+b)] of *S. williamsii* Hance leaves were determined.

### Data processing

Data were analyzed using a completely randomized design. Analysis of variance (ANOVA) was carried out to evaluate the effects of different treatments on photosynthetic parameters and [Chl(a+b)] in *S. williamsii* Hance container seedlings. When significant differences were found among the treatments, least significant difference test was conducted to detect differences among individual treatment means. All statistical analyses were performed at a significance level of *p* < 0.05. ANOVA and multiple comparisons were performed using SPSS software (version 19.0; SPSS Inc., Chicago, Illinois, USA).

## Results

### Photosynthetic rate

**Fig. 1:**
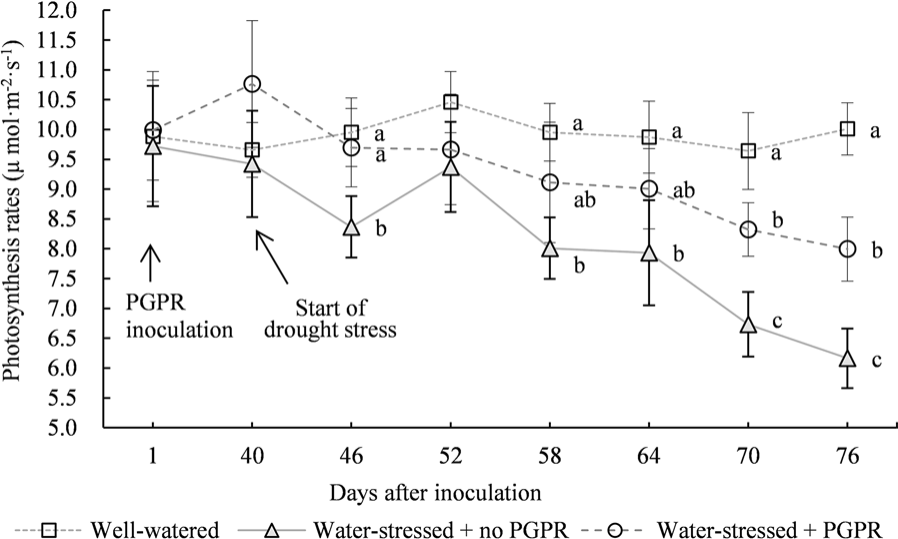
Effects of *Acinetobacter calcoaceticus* X128 inoculation on the photosynthetic rates of *Sambucus williamsii* Hance container seedlings under drought-stressed condition. Values are means of three repeating groups (twelve seedlings for each repeat group). Bars represent the standard deviation. Different letters indicate significant differences for the photosynthetic rates on the same day after inoculation at *P* < 0.05 by LSD

During the entire drought stress process, regardless of *A. calcoaceticus* X128 inoculation, *P*_n_ of *S. williamsii* Hance leaves declined, as shown in Fig. 1. At 6 days after the drought stress, *P*_n_ of uninoculated seedlings significantly declined by 15.95% compared with that under well-watered treatment (*p* < 0.05). At 12 days, relative to well-watered leaves, *P*_n_ of leaves under drought stress was reduced by 10.31%. The reduction amplitude was decreased in comparison with that in the initial phase. This result may be due to *S. williamsii* Hance forming a certain adaptation mechanism to drought stress with time. At 36 days after the drought stress, *P*_n_ of *S. williamsii* Hance leaves was reduced by 38.43% compared with that under well-watered treatment.

The average *P*_n_ values during the entire drought stress showed that X128 treatment increased *P*_n_ value by 12.99% relative to uninoculated treatment (*p* < 0.05). In the initial drought stress phase (at 6 days), X128 treatment increased *P*_n_ value by 15.90% in comparison with that under uninoculated treatment (*p *< 0.05). Thus, uninoculated *S. williamsii* Hance seedling could be easily impacted by drought stress. Subsequently, as the stress continued, *P*_n_ after X128 inoculation was elevated by 3.11%, 13.78%, 13.50%, 23.64%, and 29.73% compared with those not inoculated with X128. The difference between the two treatments gradually increased, indicating that as the drought stress duration was lengthened, the effect of X128 on inhibiting *P*_n_ reduction was gradually enhanced.

### Stomatal conductance

**Fig. 2:**
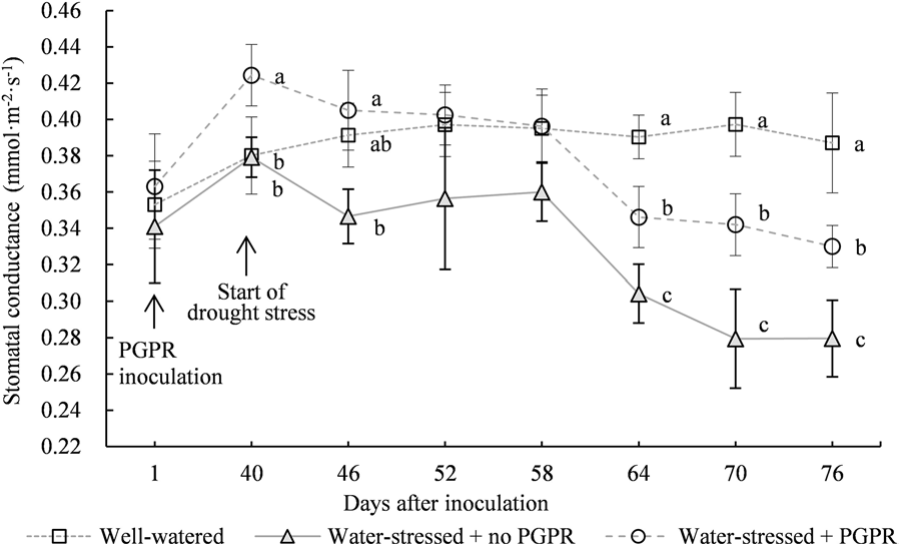
Effects of *Acinetobacter calcoaceticus* X128 inoculation on the stomatal conductance of *Sambucus williamsii* Hance container seedlings under drought-stressed condition. Values are means of three repeating groups (twelve seedlings for each repeat group). Bars represent the standard deviation. Different letters indicate significant differences for the stomatal conductance on the same day after inoculation at *P* < 0.05 by LSD

In the initial drought stress phase (at 6 days), *G*_s_ value of uninoculated *S. williamsii* Hance leaves was reduced by 11.4% due to drought stress (Fig. 2). At 12 days after continuous drought stress, *G*_s_ value of uninoculated leaves slightly increased, possibly because *S. williamsii* Hance leaves gradually adapted to the arid environment. Afterward, *G*_s_ value continued to drop. Until the final stress phase, *G*_s_ value was remarkably reduced by 27.79% compared with that under well-watered treatment (*p* < 0.05).

At 40 days after X128 inoculation, *G*_s_ value was obviously increased by 11.6% compared with that of uninoculated leaves, indicating that X128 could promote the stomatal opening of *S. williamsii* Hance leaves. Under drought stress, *G*_s_ value under X128 inoculation treatment presented a continuously declining tendency. At 6 days after the drought stress, the difference between X128 inoculation and well-watered treatment was insignificant in terms of *G*_s_ value (*p* > 0.05), but it was significantly increased by 16.8% compared with that of uninoculated drought stress treatment (*p* < 0.05). This finding indicates that X128 inoculation could relieve the inhibitory effect of drought stress on *G*_s_ value since the initial stress phase. During the entire stress period, *G*_s_ value under X128 inoculation was significantly elevated by 14.7% compared with that under uninoculated treatment (*p* < 0.05), indicating that X128 inoculation could inhibit the drought stress-induced reduction of *G*_s_.

### Intracellular CO_2_ concentration

**Fig. 3:**
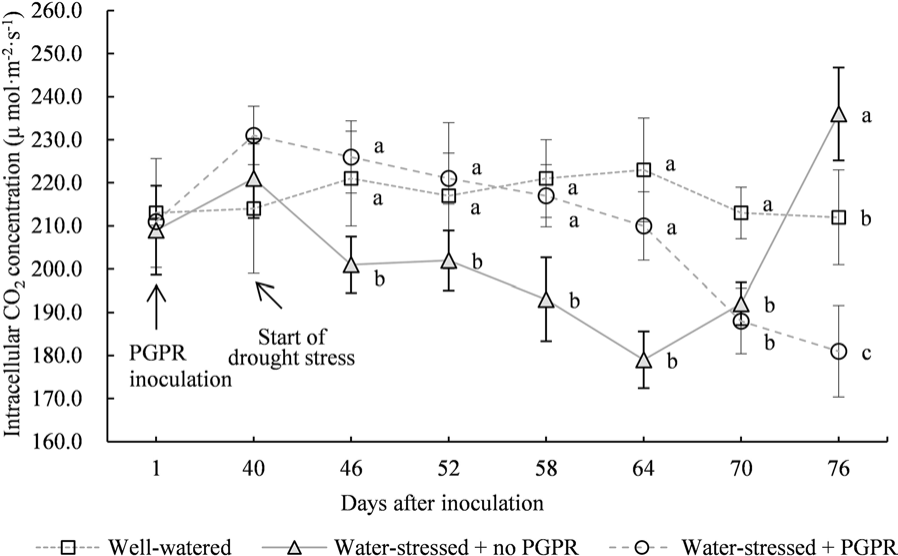
Effects of *Acinetobacter calcoaceticus* X128 inoculation on the intracellular CO_2_ concentrations of *Sambucus williamsii* Hance container seedlings under drought-stressed condition. Values are means of three repeating groups (twelve seedlings for each repeat group). Bars represent the standard deviation. Different letters indicate significant differences for the intracellular CO_2_ concentrations on the same day after inoculation at *P* < 0.05 by LSD

The effects of different treatments on *C*_i_ value at different drought stress times are shown in Fig. 3. In the initial drought stress phase (at 6 days after the drought stress treatment), in comparison with that under well-watered treatment, *C*_i_ value of uninoculated *S. williamsii* Hance leaves significantly declined by 9.05% (*p *< 0.05), indicating that the short-time drought stress could lead to reduction of the *C*_i_ value of *S. williamsii* Hance leaves. With continued stress, *C*_i_ value continued to decline, but it started increasing at 30 days. Up to the final stress phase, *C*_i_ was even significantly higher than that under well-watered treatment. At 36 days after the drought stress treatment, *C*_i_ value of walnut leaves under uninoculated drought stress treatment was elevated by 9.81% compared with that under well-watered treatment (*p* < 0.05).

During the entire stress process, the change of *C*_i_ value under X128 inoculation was not consistent with that under uninoculated treatment. In the initial drought stress phase, *C*_i_ value of leaves under X128 inoculation did not show any significant change relative to that under well-watered treatment (*p *> 0.05). However, it presented a continuously declining tendency with drought stress time. As the drought stress was continued for 36 days, *C*_i_ value under X128 treatment was significantly decreased by 15.42 (*p *< 0.05) compared with that under well-watered treatment. The level was equivalent to that at 24 days under DR drought stress treatment.

### Chlorophyll content

As drought stress was continued, the effects of different treatments on [Chl(a+b)] were different (Table 2). Before the drought stress and during the initial drought stress phase (at 10 days after the drought stress), the differences among the three treatments in terms of [Chl(a + b)] of *S. williamsii* Hance leaves were insignificant (*p* > 0.05). This finding indicates that X128 inoculation had a minor influence on [Chl(a+b)] of *S. williamsii* Hance leaves under well-watered condition and that *S. williamsii* Hance could strongly resist drought stress. With continued stress, [Chl(a+b)] under uninoculated drought stress treatment started declining. At 76 days after the inoculation (36 days after the drought stress), [Chl(a+b)] under uninoculated drought stress treatment greatly declined by 13.49% (*p* < 0.05) compared with that under well-watered treatment. Thus, drought stress could result in the decomposition or loss of chlorophyll in *S. williamsii* Hance leaves. However, X128 inoculation decreased this deficit to only 7.39%. X128 inoculation could remarkably inhibit the degradation of total chlorophyll in the final drought stress phase so that it could be basically maintained at the control level.

**Table 2:** [Chl(a+b)] (mg g^−1^) change with different experimental treatments under drought stress

Treatments	Days after inoculation				
	1	40	50	60	76
Well-watered	5.31^a^ (0.23^b^)a^c^	5.27 (0.31)a	5.35 (0.17)a	5.26 (0.13)a	5.41 (0.16)a
Water-stressed +no PGPR	5.19 (0.16)a	5.33 (0.24)a	5.24 (0.17)a	4.82 (0.10)b	4.68 (0.18)b
Water-stressed +PGPR	5.22 (0.21)a	5.29 (0.28)a	5.24 (0.22)a	5.15 (0.17)ab	5.14 (0.22)ab

^a^Values are means of three repeating groups (twelve seedlings for each repeat group)

^b^Numbers are standard deviations

^c^Different letters indicate significant differences among treatments at *P* < 0.05 by LSD

### Photosynthetic parameters after the recovery of water supply

The photosynthetic parameters of *S. williamsii* Hance seedlings bearing drought stress after their water supply was recovered for 6 days are shown in Table 3. Compared with those under well-watered conditions, the seedlings under uninoculated drought stress showed reduced *P*_n_, *G*_*s*_, and [Chl(a+b)] by 14.23%, 12.02%, and 5.86%, respectively. However, *C*_i_ was increased by 6.48%. In the final drought stress phase (at 36 days after the drought stress), *P*_n_, *G*_*s*_, and [Chl(a+b)] were reduced by 38.43%, 27.29%, and 8.37%, respectively, and *C*_i_ was increased by 11.32% relative to those under well-watered treatment. After the water supply was recovered for *S. williamsii* Hance seedlings, photosynthetic functions were partially recovered. Compared with seedlings under uninoculated drought stress treatment, seedlings under X128 inoculation showed increased *P*_n_, *G*_*s*_, and [Chl(a+b)] by 9.83%, 9.30%, and 6.85%, respectively, basically recovering to the control levels, except that *C*_i_ was slightly reduced. Therefore, under the drought stress intensity in this study, the drought stress borne by X128-inoculated *S. williamsii* Hance seedlings was irreversible.

**Table 3:** Photosynthetic parameters of *S. williamsii* Hance leaves on the 6th day after the recovery of water supply

Treatments	*P*_n_ (μmol m^−2^ s^−1^)	*G*_*s*_ (mmol m^−2^ s^−1^)	*C*_i_ (*μ*mol mol^−1^)	[Chl(a + b)] (mg g^−1^)
Well-watered	9.84^a^ (0.36^b^)a^c^	0.391 (0.017)a	212.31 (10.17)b	5.44 (0.31)a
Water-stressed + no PGPR	8.44 (0.27)a	0.344 (0.020)b	226.06 (8.59)a	4.09 (0.22)b
Water-stressed + PGPR	9.27 (0.44)ab	0.376 (0.014)a	199.34 (11.03)c	4.37 (0.14)a

^a^Data are the means of three repeating groups (twelve seedlings for each repeat group)

^b^Numbers are standard deviations

^c^Different letters indicate significant differences among treatments at *P* < 0.05 by LSD

## Discussion

### Drought stress, X128, and photosynthetic characteristics of leaves

Photosynthesis is the physiological foundation for plant growth and reflects plant growth vigor and drought resistance (Yang et al. 2014). Drought stress causes stomatal closing and a reduction of *G*_s_ and *P*_n_ (Liang et al. 2018). During the initial drought stress phase in this study, *P*_n_ value of leaves declined to a certain degree. However, as *S. williamsii* Hance seedlings adapted to the arid environment, *P*_n_ somehow increased. With continued drought stress, *P*_n_ value decreased again in the final stress phase. In their research on soybeans, Prudent et al. (2015) found that as drought stress duration is lengthened, photosynthetic rate continues to decline, which is greatly different from the conclusion drawn in the present study. This difference may be caused by the varied response modes of different plants to drought stress.

X128 inoculation under arid environment could notably repress the reduction amplitude of *P*_n_ and *G*_s_ values of *S. williamsii* Hance seedling and leaves. Except for the initial drought stress phase (at 6 days), this inhibitory effect of X128 was gradually enhanced with continued drought stress. Thus, the higher the drought stress intensity borne by the plant, the stronger the promoting effect of X128 on *P*_n_ value. Under well-watered conditions, PGPR can elevate the *P*_n_ value of plant leaves and promote dry matter accumulation (Freitas et al. 2019). However, there are few similar research reports under arid environment because different environmental conditions, such as weather, soil property, and moisture, affect microbial growth and reproduction and only PGPR are competitive and can be planted within the rhizosphere so as to exert their growth-promoting effect. The adaptation of PGPR to soil environment is a decisive factor of their functional effect (Vejan et al. 2016). X128 selected in this study was screened out from arid environment. Thus, X128 may be adaptative to arid ecological environment and influence the photosynthetic properties of *S. williamsii* Hance leaves under arid environment.

### Drought stress, X128, stomatal restriction, and non-stomatal restriction

The factors of the plant under drought stress, which caused reduced *P*_n_ value in leaves, could be mainly divided into stomatal restriction and non-stomatal restriction (Erel et al. 2015). When the drought stress was mild, *P*_n_ reduction was mainly due to the closure of some stomates (Tombesi et al. 2015). Under severe drought stress, the activities of some enzymes participating in carbon fixation in the chloroplast were inhibited due to moisture loss of chloroplast and cells. Furthermore, *P*_n_ value was reduced mainly due to non-stomatal factors (Signarbieux and Feller 2011). *C*_i_ and *G*_s_ are criteria for stomatal or non-stomatal factors causing *P*_n_ reduction. *C*_i_ value was reduced because of stomatal restriction, whereas it was elevated due to non-stomatal restriction. When both stomatal and non-stomatal restrictions existed, the change law of *C*_i_ value depended on the dominant factor (Reed and Loik 2016; Bellasio et al. 2018). The present study showed that in the initial drought stress phase, *P*_n_ reduction of *S. williamsii* Hance leaves was accompanied by a reduction of *G*_s_ and *C*_i_, indicating that *P*_n_ reduction was mainly restricted by stomatal factors. However, as the drought stress duration was lengthened, *G*_s_ continued to decline, whereas *C*_i_ gradually increased after declining initially. This finding indicates that *P*_n_ reduction started being restricted by non-stomatal factors besides stomatal factors, photosynthetic activity of mesophyll cells was degraded, and photosynthetic organ structure was injured. Furthermore, after the water supply was recovered, *P*_n_ failed to recover to the level under well-watered condition. However, after X128 inoculation, *C*_i_ value continuously decreased during the entire drought stress period. Therefore, under the drought stress intensity in this study, *P*_n_ reduction of *S. williamsii* Hance leaves was closely associated with stomatal factors and was always restricted by stomatal factors but not non-stomatal factors, even during the final stress phase. This finding indicates that under the stress intensity in this study, X128 inoculation could reduce the structural injury of photosynthetic organs of leaves, mitigate the restrictions caused by non-stomatal factors to *P*_n_ value of leaves under water stress, and contribute to the rapid recovery of photosynthetic functions after the recovery of water supply treatment.

### Drought stress, X128, and [Chl(a+b)]

Leaf photosynthesis is generally correlated with chlorophyll content (Kanwal et al. 2017). Results of several studies indicate that water stress adversely affects the chlorophyll content, and the higher the drought intensity is, the greater the chlorophyll content decreases. (Nageswara Rao et al. 2001; Fotovat et al. 2007). The results of this study indicate that the leaf chlorophyll content significant decreased under drought stress compared with that under well-watered conditions; this result confirmed the findings of previous studies. In the initial and middle drought stress phases in this study, [Chl(a+b)] in *S. williamsii* Hance leaves did not significantly change regardless of X128 inoculation. This finding indicates the strong adaptation ability of *S. williamsii* Hance to drought stress to a certain degree. Nevertheless, as the drought stress duration was lengthened, [Chl(a+b)] in uninoculated leaves was reduced to a certain degree, which might be related to the destructive effect of water stress-induced membrane lipid peroxidation of chloroplast. After the water supply was recovered, [Chl(a+b)] of uninoculated *S. williamsii* Hance leaves failed to recover to the well-watered level, indicating that *S. williamsii* Hance experienced irreversible drought stress-induced injury. Ying et al. (2015) believed that as the drought stress intensity increases, [Chl(a+b)] in *Camptotheca acuminata* seedlings continues to decrease, similar to the study result regarding elderberries. If the stability of [Chl(a+b)] can be guaranteed under moderate drought stress, it will be conducive to inhibiting *P*_n_ reduction and enhancing plant stress resistance (Nageswara Rao et al. 2001). After X128 inoculation, [Chl(a+b)] of *S. williamsii* Hance leaves did not significantly change under moderate drought stress, proving that X128 inoculation improved the adaptation ability of *S. williamsii* Hance to arid environment to a certain degree.

The X128 selected in this study could generate CTKs of certain concentrations. CTKs can promote the opening of stomates and elevate photosynthetic rate (Arkhipova et al. 2007; Vysotskaya et al. 2003). CTKs can inhibit the loss of total chlorophyll to a certain extent (Abbasi et al. 2011). In the present study, [Chl(a+b)] of *S. williamsii* Hance leaves was somehow reduced during the final drought stress phase but was suppressed by X128 inoculation. Therefore, reducing the loss of total chlorophyll and regulating the opening and closure of stomates by elevating CTK content in plant leaves may be a mechanism of X128 in relieving the inhibitory effect of drought stress on *P*_n_ value of plant leaves. PGPR inoculation under arid environment can effectively inhibit the reduction of *P*_n_ and improve the plant’s adaptation ability to arid environment. These findings will be of outstanding significance to forestation and forest tending on arid mountains.

## Data Availability

The authors declare that all the data and materials used in this study are available.
